# Effect of intraarticular drug injection in patients with temporomandibular joint disorders with limited mouth opening: a system review and network meta-analysis

**DOI:** 10.22514/jofph.2025.041

**Published:** 2025-12-12

**Authors:** Siyu Zou, Yue Yue, Xinyu Wang, Qinglan Liu

**Affiliations:** ^1^School of Stomatology, Nanjing Medical University, 210029 Nanjing, Jiangsu, China; ^2^Second Clinical Medical College, Nanjing Medical University, 210029 Nanjing, Jiangsu, China; ^3^Department of Oral Mucosal Disease, The Affiliated Stomatology Hospital of Nanjing Medical University, State Key Laboratory Cultivation Base of Research, Prevention and Treatment for Oral Diseases, Jiangsu Province Engineering Research Center of Stomatological Translational Medicine, 210029 Nanjing, Jiangsu, China

**Keywords:** Articular cavity injection of drugs, Network meta-analysis, Platelet-rich fibrin, Temporomandibular joint disorders

## Abstract

**Background**: This network meta-analysis (NMA) aims to evaluate the comparative efficacy of 13 intra-articular pharmacologic interventions in improving maximal mouth opening (MMO), pain relief, and functional recovery in patients with temporomandibular disorders (TMD) and restricted jaw mobility. **Methods**: A comprehensive literature search was conducted in PubMed, Embase, Cochrane Library, and Web of Science to identify randomized controlled trials (RCTS) evaluating the effects of 13 intra-articular drug injections in patients with limited mouth opening TMD. The methodological quality of the included studies was assessed using the risk of bias (ROB) tool, and data were independently extracted by two researchers. Primary outcomes included maximum mouth opening (MMO) and pain intensity; secondary outcomes included joint lateral and protrusive movements. **Results**: A total of 38 RCTs involving 1533 TMD patients were included. Thirteen different agents (including HA: hyaluronic acid, PRF: platelet-rich fibrin, PDGF: platelet-derived growth factor, PRP: platelet-rich plasma, MOR: morphine, LA: local anesthetic, MA: micro-fragment fat, TRA: tramadol, SAL: Saline, GC: glucocorticoid, GLU: glucose, NS: non-steroidal, and ARTH: arthrocentesis only) were evaluated. NMA results showed that PRF injection after arthrocentesis significantly improved the maximum temporomandibular joint opening (surface under the cumulative ranking curve (SUCRA): 99.1%) compared with arthrocentesis alone. MA injection following arthrocentesis was most effective for pain reduction (SUCRA: 84.7%), followed by PRF (SUCRA: 78.2%). PRF also led to significant improvements in lateral jaw movement (SUCRA: 95.5%) and protrusive movement (SUCRA: 63.5%) compared to arthrocentesis alone. **Conclusions**: Based on the network ranking chart, PRF injection after arthrocentesis offers the greatest benefits for functional recovery in TMD patients. However, additional rigorous literature is required to validate this assertion. **Clinical Trial Registration**: Registration number is INPLASY202450107.

## 1. Introduction

Temporomandibular joint disorder (TMD) encompasses a group of conditions affecting the temporomandibular joints and associated masticatory muscles. It is primarily characterized by joint and muscle pain, limited mouth opening [1], and functional limitations of the jaw. TMD significantly impairs patients’ quality of life and affects millions of individuals worldwide, ranking as the second most common chronic pain disorder after back pain [2, 3].

Arthrocentesis is a minimally invasive treatment option for TMJ (Temporomandibular Joint), involving lavage of the upper and lower joint compartments to remove inflammatory mediators. While the superior compartment is more frequently irrigated due to its spatial accessibility [4], selective administration of pharmaceuticals (*e.g.*, platelet-rich plasma or hyaluronate) into the inferior compartment has shown enhanced efficacy in cases with advanced disc displacement or adhesions, as demonstrated by recent clinical trials [5].

Arthrocentesis has been shown to be a highly effective surgical method with a high success rate and favorable cost-benefit ratio. Beyond arthrocentesis, intra-articular injection represents a commonly employed non-surgical strategy aimed at alleviating pain and enhancing jaw function. Numerous agents have been reported in the literature for intra-articular administration, including hyaluronic acid (HA), glucocorticoids, local anesthetics, morphine, tramadol, saline, glucose, and non-steroidal drugs (NSAIDs). HA is widely used for its nutritive, protective, lubricating, and anti-inflammatory effects, playing an important role in maintaining TMJ homeostasis. Asadpour N *et al*. [6] found that HA injection significantly improved maximum mouth opening (MMO) of Temporomandibular joint osteoarthritis (TMJOA) over a short-term period. Glucocorticoids, owing to their potent anti-inflammatory effects, have long been used to relieve pain in TMD. Studies have shown that intra-articular glucocorticoids injection can significantly relieve the clinical symptoms of TMD [7]. According to Lubecka *et al*. [4], there is insufficient scientific evidence supporting the effect of intra-articular local anesthesia on the range of jaw motion, and injections are considered only temporary analgesic measures. Gopalakrishnan *et al*. [8] evaluated the efficacy of intra-articular analgesic drugs, including opioids and NSAIDs following TMJ puncture, reporting enhanced outcomes with morphine and fentanyl, and superior efficacy of tramadol compared to COX-2 (Cyclooxygenase-2) inhibitors. Liapaki *et al*. [9] showed that normal saline injection could significantly improve TMJ pain and MMO. In addition to these agents, platelet-rich plasma (PRP) and platelet-derived growth factor (PDGF) have emerged as promising autologous biologics. A study by Haddad *et al*. [10] demonstrated that PRP injections significantly enhanced joint mobility and reduced pain, with therapeutic effects persisting for up to 12 months post-treatment.

Platelet-rich fibrin (PRP), a second-generation blood concentrate characterized by a dense three-dimensional fibrin matrix, offers notable advantages in regenerative medicine. Its preparation involves a one-step centrifugation process and does not require anticoagulations or external activators. Recent studies have reported the clinical benefit of injection of liquid platelet-rich fibrin (I-PRF) as an adjunctive therapy after joint puncture in TMD patients [11, 12, 13, 14]. I-PRF appeared to be superior in long-term efficacy, with PRF providing more encouraging results in improving MMO after 3 and 6 months. In parallel, Micro-fragmentation of adipose tissue (MA)—a method well established in orthopedic applications for its enhanced stem cell preservation—has recently been introduced in TMJ therapy. Lubecka K *et al*.’s [15] study showed that MA injection significantly improve TMJ pain and functional problems.

Despite the promising results of various intra-articular treatments—such as hyaluronic acid (HA), platelet-rich plasma (PRP), platelet-rich fibrin (PRF), glucocorticoids, and other medications—their comparative efficacy remains insufficiently defined. Network meta-analysis (NMA), a robust methodological framework, enables both direct and indirect comparisons across multiple treatment options, offering essential guidance for clinical decision-making in pharmacotherapy. In this study, we conducted a comprehensive NMA that included 13 intra-articular interventions drawn from 38 randomized trials (RCTs) with 1533 patients diagnosed with Mouth-opening-restricted TMD. This analysis aimed to systematically rank the effectiveness of each treatment and identify the optimal intervention strategy.

## 2. Materials and methods

This systematic review was conducted in strict accordance with the Preferred Reporting Items for Systematic Reviews and Meta-Analyses (PRISMA) guidelines (see the section **Supplementary material 1**) and has been duly registered with the International Platform of Registered Systematic Review and Meta-analysis Protocols (INPLASY) under the registration number INPLASY202450107.

### 2.1 Search strategy

We conducted a network meta-analysis to compare the effectiveness of different intra-articular drug injections in patients with TMD. A comprehensive search of four electronic databases (PubMed, Embase, Cochrane Library, Web of Science) was performed from their inception until 20 January 2025. The search strategy was developed based on the PICOS framework: (P) Participants: patients with TMD and limited mouth opening; (I) Intervention: intra-articular drug injection; (C) Comparator: joint puncture without drug administration; (O) Outcomes: pain index and joint mobility; (S) Study design: randomized controlled trial. Detailed search strategies are presented in Table 1 (Pubmed strategy shown as an example).

**Table 1.** Search strategy on PubMed.

#1	Temporomandibular disorders[MeSH Terms]
#2	((((((((((((((((((((((Disorder, Temporomandibular Joint[Title/Abstract]) OR (Disorders, Temporomandibular Joint[Title/Abstract])) OR (Joint Disorder, Temporomandibular[Title/Abstract])) OR (Joint Disorders, Temporomandibular[Title/Abstract])) OR (Temporomandibular Joint Disorder[Title/Abstract])) OR (TMJ Disorders[Title/Abstract])) OR (Disorder, TMJ[Title/Abstract])) OR (Disorders, TMJ[Title/Abstract])) OR (TMJ Disorder[Title/Abstract])) OR (Temporomandibular Disorders[Title/Abstract])) OR (Disorder, Temporomandibular[Title/Abstract])) OR (Disorders, Temporomandibular[Title/Abstract])) OR (Temporomandibular Disorder[Title/Abstract])) OR (Temporomandibular Joint Diseases[Title/Abstract])) OR (Disease, Temporomandibular Joint[Title/Abstract])) OR (Diseases, Temporomandibular Joint[Title/Abstract])) OR (Joint Disease, Temporomandibular[Title/Abstract])) OR (Joint Diseases, Temporomandibular[Title/Abstract])) OR (Temporomandibular Joint Disease[Title/Abstract])) OR (TMJ Diseases[Title/Abstract])) OR (Disease, TMJ[Title/Abstract])) OR (Diseases, TMJ[Title/Abstract])) OR (TMJ Disease[Title/Abstract])
#3	(#1) OR (#2)
#4	Injections, Intra-Articular[MeSH Major Topic]
#5	((((((((((((((Injections, Intra-Articular[Title/Abstract]) OR (Intra-articular injection[Title/Abstract])) OR (Injection, Intra-Articular[Title/Abstract])) OR (Intra-Articular Injection[Title/Abstract])) OR (Intraarticular Injection[Title/Abstract])) OR (Injection, Intraarticular[Title/Abstract])) OR (Intraarticular Injections[Title/Abstract])) OR (Intra-Articular Injections[Title/Abstract])) OR (Injections, Intraarticular[Title/Abstract])) OR (Intra Articular Injection[Title/Abstract])) OR (Articular Injection, Intra[Title/Abstract])) OR (Articular Injections, Intra[Title/Abstract])) OR (Injection, Intra Articular[Title/Abstract])) OR (Injections, Intra Articular[Title/Abstract])) OR (Intra Articular Injections[Title/Abstract])
#6	(#4) OR (#5)
#7	((((((((mouth opening restriction[Title/Abstract]) OR (limitation of mouth opening[Title/Abstract])) OR (limited mouth opening[Title/Abstract])) OR (restriction of mouth opening[Title/Abstract])) OR (limited opening[Title/Abstract])) OR (limitation of mouth[Title/Abstract])) OR (mouth opening limit[Title/Abstract])) OR (placket restrained[Title/Abstract])) OR (mouth opening limitation[Title/Abstract])

### 2.2 Inclusion criteria

(1) Unilateral TMD, localized temporomandibular joint pain; (2) A Wilkes classification of stage 3 or above, as confirmed by magnetic resonance imaging (MRI) or computed tomography (CT), in conjunction with clinical evaluation; (3) Intra-articular drug injection following joint puncture, or joint puncture alone without additional drug injection; (4) Studies published in peer-reviewed journals from database inception to January 2025. (5) Randomized controlled trials (RCTs) reporting the effects of intra-articular drug injections on TMD; (6) Assessment of pain using the visual analogue scores (VAS) and maximal mouth opening (MMO) before and after treatment; (7) Evaluation of temporomandibular joint function, including joint lateral movement (LM) and protrusive movement (PM).

### 2.3 Exclusion criteria

(1) Autoimmune disorders, patients with temporomandibular joint trauma, significant mechanical obstruction preventing mouth opening, acute bursitis, benign and malignant TMJ tumors, neurological disorders, hematological diseases, coagulation abnormalities, and a history of allergies or anaphylaxis; (2) Retrospective non-comparative case series, medical record reviews, conference abstracts, historical articles, editorials, letters, literature reviews, and experimental research; (3) Studies with incomplete or insufficiently unreported outcomes. (4) No language restrictions were applied to the selection of studies.

### 2.4 Study selection

The process of literature screening and selection was conducted using Endnote, a professional reference management software. Initially, duplicates records, non-randomized controlled trials, retrospective studies, conference presentations, study protocols, and correspondence were screened. Subsequently, abstracts were reviewed to determine eligibility according to the predefined inclusion criteria. Full texts were then retrieved for detailed assessment, and studies were categorized as included or excluded accordingly. The screening process was independently performed by two reviewers. Discrepancies between reviewers were resolved through discussion to reach a consensus.

### 2.5 Data extraction

Data extraction was performed using a standardized form encompassing six pre-defined items, which were recorded in structured tables. The categories included: (1) author, (2) year of publication, (3) country of study, (4) sample size, (5) details of the pharmacological intervention, and (6) outcomes resulting from the intervention.

### 2.6 Literature quality assessment

The methodological quality of the included RCTs was assessed using the Cochrane Collaboration’s Risk of Bias (ROB) tool. This evaluation covered seven critical domains: random sequence generation, allocation concealment, blinding of participants and personnel, blinding of outcome assessment, incomplete outcome data, selective reporting, and other potential biases. Two independent reviewers conducted the assessment, and each domain was rated as having a low, high, or unclear risk of bias based on established criteria. Any disagreements were resolved through consensus discussions [16].

### 2.7 Data analysis

In research that incorporates pharmacological interventions, all variables were treated as continuous and presented as mean ± standard deviation (SD). The continuous variables from the study will be articulated as either the mean difference (MD), which represents the absolute disparity between the two groups (calculated as the difference between treatment and control groups using a consistent metric), or the standardized mean difference (SMD), which is derived from the difference in outcomes between the mean values of the groups divided by the standard deviation among subjects. Both metrics will be accompanied by 95% confidence intervals (CI) for thorough analysis. Given the anticipated heterogeneity across studies, a random-effects model was employed for our analysis rather than a fixed-effects model [17]. The analysis was conducted using Stata software (version 15.1, StataCorp LLC, College Station, TX, USA), following the PRISMA-NMA guidelines by employing Markov chain Monte Carlo simulation techniques within a Bayes-based framework for the purpose of NMA aggregation and examination [18, 19]. To assess the consistency between direct and indirect evidence, a node-splitting method was used, with a *p*-value > 0.05 indicating acceptable consistency [20]. The consistency model was confirmed valid. Network diagrams were generated to illustrate the relationships among interventions, where each node represented a specific drug or control condition, and the connecting lines indicated direct comparisons. The dimensions of each node, along with the thickness of the connecting lines were proportional to the number of studies included [21]. The hierarchy of the interventions was summarized and reported using a P score, which serves as a frequency analog to the cumulative ranking curve (SUCRA) value and quantifies the level of certainty regarding the superiority of one treatment over another, averaged across all competing treatments. P scores range from 0 to 1, with 1 denoting absolute certainty and 0 signifying the absence of certainty. While the P scores or SUCRA can be effectively reinterpreted as a percentage indicating the efficacy or acceptability of a pharmacological intervention, it is critical to interpret these scores with caution unless clinically significant differences between the interventions are evident. To explore potential biases arising from smaller studies that might contribute to publication bias in NMA, a network funnel plot was constructed and visually inspected for symmetry.

## 3. Results

### 3.1 Study identification and selection

Initially, a total of 5632 documents were identified through electronic database research, with an additional 13 documents retrieved manually. After the removal of duplicates, 4792 documents remained for screening based on titles and abstracts. Of these, 4365 documents were excluded. The remaining 427 full-text articles were assessed for eligibility, resulting in the exclusion of 389 studies for various reasons, including the classification as non-randomized controlled trials, incomplete data, conference proceedings, and non-adherence to the interventions outlined in this review. Ultimately, 38 studies met all inclusion criteria and were included in the final analysis (Fig. 1).

**Fig. 1.** PRISMA flow diagram of study selection. Of 5632 records identified, 38 randomized controlled trials (RCTs) met inclusion criteria.

### 3.2 Quality evaluation of the selected studies

The risk-of-bias analysis across 38 studies revealed the following findings: Random sequence generation demonstrated the highest quality, with all studies rated as low risk, indicating well-documented and appropriate randomization methods. Similarly, selective reporting and other biases were rated as low risk at 76% and 82%, respectively. However, significant concerns were identified about allocation concealment. 18% of studies were classified as high risk (*e.g.*, Akhilesh Kumar Singh 2019 lacked description of allocation concealment methods) and 35% as unclear (*e.g.*, insufficient methodological details in Preeti Sharma 2023). In blinding implementation, 13% of studies exhibited high risk in both participant/personnel blinding (*e.g.*, open-label design in Marijus Leketas 2022) and outcome assessor blinding (*e.g.*, non-blinded assessors in Jose-Maria Oliveras-Moreno 2008). For incomplete outcome data, 18% of studies were high risk (*e.g.*, Songül Cömert Kiliç 2015 reported high attrition rates without appropriate handling), while other biases included baseline imbalances (D Manfredini 2012) and funding conflicts (Wynand Francois Louw 2018) in 11% of cases. In summary, while the studies demonstrated overall strengths in randomization procedures and outcome reporting, critical methodological limitations were evident in allocation concealment, blinding practices, and data completeness. Sensitivity analyses are recommended for high-risk studies (*e.g.*, Sha-Sha Liu, Li-Li Xu 2023). Future research should prioritize double-blinding protocols, explicit allocation concealment descriptions, and standardized data reporting to enhance methodological reliability and minimize bias (Fig. 2).

**Fig. 2.** Risk of bias summary across 38 included studies, assessed using the Cochrane ROB tool. Domains: (1) random sequence generation, (2) allocation concealment, (3) blinding of participants/personnel, (4) blinding of outcome assessment, (5) incomplete outcome data, (6) selective reporting, (7) other biases. Green: low risk; yellow: unclear risk; red: high risk.

### 3.3 Attributes of the studies under review

38 RCTs, encompassing 1533 patients diagnosed with TMD were included in this review. Drug injection interventions include hyaluronic acid (18 studies) [22, 23, 24], injection of platelet-rich fibrin (3 studies) [25, 26, 27], platelet-derived growth factor (2 studies) [28, 29], platelet-rich plasma (9 studies) [30], morphine (1 study) [31], and the following: Local anesthesia (3 studies) [32], micro fragmental fats (1 study) [33], tramadol (1 study) [31], saline (9 studies) [34], glucocorticoids (11 studies) [7, 35, 36], glucose (5 studies) [37, 38, 39], nonsteroidal drugs (2 studies) [30, 40], and other drugs. Only arthrocentesis was performed (2 studies) [41, 42]. Regarding outcome measures, 33 studies reported MMO as an outcome measure, 36 assessed VAS, 9 reported LM, and 11 reported PM. A detailed summary of the included studies and their characteristics is provided in Table 2.

**Table 2.** Characteristics of the studies included in the meta-analysis.

Author	Country	Year	Total patients	Treatment	Outcome
Ravina Dharamsi	India	2022	40	Arth + HA Arth + GC	MMO, VAS
Ugur Karadayi	Turkey	2021	36	Arth Arth + I-PRF	MMO, VAS
Preeti Sharma	India	2023	14	Arth + PRP Arth + I-PRF	MMO, VAS, PM, LM
T Bjørnland	Norway	2007	40	Arth + HA Arth + GC	MMO, VAS, PM, LM
Gözde Işık	Turkey	2022	36	Arth Arth + I-PRF	MMO, PM, LM
Saubhik Dasukil	India	2022	90	Arth Arth + PRP Arth + HA	MMO, VAS
Nazanin Asadpour	Iran	2022	30	Arth + PRP Arth + HA Arth + PRP + HA	MMO, VAS, PM, LM
Zeynep Bayramoglu	Turkey	2023	30	Arth Arth + NS	MMO, VAS
Zeliha Kapusuz Gencer	Turkey	2014	100	Arth + HA Arth + GC Arth + NS Arth + SAL	VAS
D Manfredini	Italy	2012	36	Arth Arth + GC Arth + HA	MMO, VAS
Ivan Solani Martins	Brazil	2023	24	Arth + GC Arth + SAL	MMO, VAS
Wael Abbadi	Syria	2022	22	Arth + PRP Arth	MMO, VAS
Marijus Leketas	Lithuania	2022	77	Arth + SAL Arth + HA Arth + PRGF	MMO, VAS
Jose-Maria Oliveras-Moreno	Spain	2008	35	Arth + NS Arth + HA	VAS
Songül Cömert Kiliç	Turkey	2015	30	Arth + GLU Arth	MMO, VAS, PM, LM
S Cömert Kiliç	Turkey	2016	26	Arth + GLU Arth + SAL	MMO, VAS, PM, LM
Martín Fernández-Ferro	Spain	2017	100	Arth + PRGF Arth + HA	MMO, VAS
Onur Yilmaz	Turkey	2019	36	Arth + HA Arth + SAL	MMO, VAS
Songül Cömert Kiliç	Turkey	2016	25	Arth + GC Arth	MMO, VAS, PM, LM
Wynand Francois Louw	Canada	2018	54	Arth + GLU Arth + GLU + LA	VAS
Göran Isacsson	Sweden	2019	54	Arth + GC Arth + SAL	VAS
Sara Bergstrand	Norway	2019	37	Arth + SAL Arth + HA	VAS, PM, LM
Bruno Macedo De Sousa	Spain	2020	80	Arth + GC Arth + HA Arth + PRP Arth	MMO, VAS
Tikaram Gurung	India	2017	20	Arth + HA Arth	MMO, VAS
Surya Udai Singh	India	2022	20	Arth + GC Arth	MMO, VAS
Reza Tabrizi	Iran	2014	60	Arth + GC Arth	MMO, VAS
Sha-Sha Liu, Li-Li Xu	China	2023	65	Arth + HA Arth + PRP	MMO, VAS
J.J.R. Huddleston Slater	Netherlands	2012	28	Arth + GC Arth	MMO, VAS
Harsha Gorrela	India	2016	62	Arth + HA Arth	MMO, VAS, PM
Akhilesh Kumar Singh1	India	2019	24	Arth + PRP Arth	MMO, VAS
Akash Rajput1	India	2020	24	Arth + PRP Arth	MMO, VAS, PM
Shiju Mathew Jacob	India	2021	45	Arth + PRP Arth + HA Arth	MMO, VAS, PM, LM
Salvatore Sembronio	Italy	2021	40	Arth + MA Arth + HA	MMO, VAS
Rawand Mustafa	Turkey	2018	18	Arth + GLU Arth + SAL	MMO, VAS
Geerten-Has E Tjakkes	Netherlands	2007	19	Arth + LA Arth + SAL	MMO, VAS
Hamida Refai	Egypt	2011	12	Arth + GLU + LA Arth + LA	MMO
A. Sipahi	Turkey	2015	20	Arth Arth + MOR Arth + TRA	MMO, VAS
Serap Moroğlu OZDAMAR	Turkey	2016	24	Arth + HA Arth	MMO, VAS

*Note: Arth: arthrocentesis; HA: hyaluronic acid; I-PRF: liquid platelet rich fibrin; PRP: platelet rich plasma; MOR: morphine; LA: local anesthetics; MA: Microfragmented Adipose; TRA: tramadol; SAL: saline; GC: glucocorticoid; GLU: glucose; NS: non-steroidal; LM: Lateral movement; PM: Protrusive movement; PRGF: Plasma Rich in Growth Factors; MMO: Maximal Mouth Opening; VAS: Visual Analog Scale.*

### 3.4 Network meta-analysis

The complete NMA charts were presented in Figs. 3,4,5,6.

**Fig. 3.** Network geometry and cumulative ranking plots for maximal mouth opening (MMO). (A) Network geometry of interventions for maximum mouth opening (MMO). Each node represents a treatment: Node size is proportional to the number of patients receiving each intervention, and line thickness indicates the number of direct comparisons between treatments. Gray dashed lines denote indirect comparisons. (B) Ranking of interventions for improving maximum mouth opening (MMO) based on surface under the cumulative ranking curve (SUCRA) values. A = Arth + HA; B = Arth + I-PRF; C = Arth + PDGF; D = Arth + PRP; E = Arth; F = Arth + MOR; G = Arth + LA; H = Arth + MA; I = Arth + TRA; J = Arth + SAL; K = Arth + GC; L = Arth + GLU; M = Arth + GLU + LA; N = Arth + NS. Arth: arthrocentesis; PRP: platelet rich plasma; PRGF: Plasma Rich in Growth Factors; I-PRF: liquid platelet rich fibrin; HA: hyaluronic acid; NS: non-steroidal; GLU: glucose; LA: local anesthetics; GC: glucocorticoid; SAL: saline; TRA: tramadol; MA: Microfragmented Adipose; MOR: morphine.

**Fig. 4.** Network geometry and cumulative ranking plots for Visual Analogue Scale (VAS). (A) Network geometry of interventions for Visual Analogue Scale (VAS). Each node represents a treatment: Node size is proportional to the number of patients receiving each intervention, and line thickness indicates the number of direct comparisons between treatments. Gray dashed lines denote indirect comparisons. (B) Ranking of interventions for reducing Visual Analogue Scale (VAS) based on surface under the cumulative ranking curve (SUCRA) values. A = Arth + HA; B = Arth + I-PRF; C = Arth + PDGF; D = Arth + PRP; E = Arth; F = Arth + MOR; G = Arth + LA; H = Arth + MA; I = Arth + TRA; J = Arth + SAL; K = Arth + GC; L = Arth + GLU; M = Arth + GLU + LA; N = Arth + NS. Arth: arthrocentesis; PRP: platelet rich plasma; PRGF: Plasma Rich in Growth Factors; I-PRF: liquid platelet rich fibrin; HA: hyaluronic acid; NS: non-steroidal; GLU: glucose; LA: local anesthetics; GC: glucocorticoid; SAL: saline; TRA: tramadol; MA: Microfragmented Adipose; MOR: morphine.

**Fig. 5.** Network geometry and cumulative ranking plots for Lateral movement (LM). (A) Network geometry of interventions for Lateral movement (LM). Each node represents a treatment: Node size is proportional to the number of patients receiving each intervention, and line thickness indicates the number of direct comparisons between treatments. Gray dashed lines denote indirect comparisons. (B) Ranking of interventions for improving Lateral movement (LM) based on surface under the cumulative ranking curve (SUCRA) values. A = Arth + HA; B = Arth + I-PRF; C = Arth + PDGF; D = Arth + PRP; E = Arth. Arth: arthrocentesis; PRP: platelet rich plasma; I-PRF: liquid platelet rich fibrin; HA: hyaluronic acid; GC: glucocorticoid.

**Fig. 6.** Network geometry and cumulative ranking plots for Protrusive movement (PM). (A) Network geometry of interventions for Protrusive movement (PM). Each node represents a treatment: Node size is proportional to the number of patients receiving each intervention, and line thickness indicates the number of direct comparisons between treatments. Gray dashed lines denote indirect comparisons. (B) Ranking of interventions for improving Protrusive movement (PM) based on surface under the cumulative ranking curve (SUCRA) values. A = Arth + HA; B = Arth + I-PRF; C = Arth + PDGF; D = Arth + PRP; E = Arth. Arth: arthrocentesis; PRP: platelet rich plasma; I-PRF: liquid platelet rich fibrin; HA: hyaluronic acid; GC: glucocorticoid.

#### 3.4.1 Maximal mouth opening (MMO)

The *p* values associated with direct and indirect comparisons across all studies were evaluated for consistency and inconsistency. Most *p* values exceeded 0.05, suggesting that the consistency effect among the studies was deemed acceptable (**Supplementary Table 1**).

The results of NMA demonstrated that arthrocentesis + injection liquid platelet-rich fibrin (I-PRF) (MD = 15.84, 95% CI: (9.60, 22.08)) was significantly more effective than arthrocentesis alone in improving MMO in TMD patients. Specifically, I-PRF demonstrated superior efficacy over arthrocentesis with platelet-rich plasma (PRP) (MD = 12.85, 95% CI: (6.24, 19.46)), morphine (MOR) (MD = 14.04, 95% CI: (0.91, 27.17)), local anesthetic (LA) (MD = 15.64, 95% CI: (1.49, 29.78)), hyaluronic acid (HA) (MD = 15.96, 95% CI: (9.04, 22.87)), tramadol (TRA) (MD = 17.04, 95% CI: (3.76, 30.32)), glucocorticoids (GC) (MD = 16.44, 95% CI: (9.14, 23.74)), saline (SAL) (MD = 17.24, 95% CI: (7.67, 26.80)), and glucose (GLU) (MD = 19.45, 95% CI: (7.14, 31.76)). All comparisons exhibited statistically significant differences, underscoring the superior clinical efficacy of I-PRF in TMJ management (see **Supplementary Table 2**). Ranking the probability of different drug injections improving MMO differences, arthrocentesis + I-PRF ranked first among SUCRA (SUCRA = 99.1%, Fig. 3B).

#### 3.4.2 Visual analogue scale (VAS)

The *p* values obtained from direct and indirect comparisons across all studies were evaluated for consistency and inconsistency. Most *p* values exceeded 0.05, indicating satisfactory consistency within the network model (**Supplementary Table 3**).

NMA demonstrated that Arth + MA (MD = −4.31, 95% CI: (−7.33, −1.30)), Arth + PRF (MD = −3.50, 95% CI: (−5.77, −1.23)), Arth + PRP (MD = −2.75, 95% CI: (−4.24, −1.27)), Arth + HA (MD = −2.51, 95% CI: (−3.77, −1.26)), Arth + GC (MD = −1.89, 95% CI: (−3.24, −0.55)), and Arth alone (MD = −1.66, 95% CI: (−3.06, −0.26)) exhibited statistically significant superiority over Arth + SAL in alleviating patient pain. No significant differences were observed in pairwise comparisons among the remaining interventions (**Supplementary Table 4**). Arthrocentesis + MA ranked first among SUCRA (SUCRA = 84.7%, as shown in Fig. 4B).

#### 3.4.3 Lateral movement (LM)

The consistency between direct and indirect comparisons was evaluated for all included studies. Most *p* values exceeded 0.05, indicating an acceptable level of consistency within NMA mode (**Supplementary Table 5**).

NMA showed that Arth + I-PRF (MD = 0.91, 95% CI: (0.31, 1.50)), and Arth + PDGF (MD = 0.33, 95% CI: (0.03, 0.62)) were significantly superior to Arth + HA in improving the transverse movement function of TMJ. Additionally, Arth + I-PRF (MD = 0.89, 95% CI: (0.55, 1.23)) also outperformed Arth + PRP in enhancing transverse movement function of TMJ. No significant differences were observed in pairwise comparisons among the remaining interventions (**Supplementary Table 6**). Ranking the probability of different drug injections in improving patients’ lateral movement, joint puncture + I-PRF ranked first among SUCRA (SUCRA = 95.5%, Fig. 5B).

#### 3.4.4 Protrusive movement (PM)

Consistency assessment for both direct and indirect comparisons across most studies yielded *p*-values greater than 0.05, confirming acceptable consistency for the NMA model (**Supplementary Table 7**). NMA indicated no statistically significant differences in pairwise comparisons among the interventions (**Supplementary Table 8**). Arthrocentesis + I-PRF ranked first among SUCRA (SUCRA = 63.5%, Fig. 6B) in the probability ranking of different drug injections in improving patients’ anterior protrusive movement.

### 3.5 Publication bias test

Individual funnel plots were constructed for all outcome measures to examine possible publication bias. A visual examination of the funnel plot did not reveal any significant publication bias (Fig. 7).

**Fig. 7.** Funnel plots assessing potential publication bias for (A) MMO, (B) VAS, (C) LM, and (D) PM outcomes. Each point represents a study’s effect size (x-axis: mean difference) against its precision (y-axis: standard error). Symmetrical distribution around the pooled effect (vertical dashed line) suggests low risk of bias. Asymmetry may indicate missing studies or heterogeneity. Red dotted lines denote 95% confidence limits. In Groups A and B: A = Arth + HA; B = Arth + I-PRF; C = Arth + PDGF; D = Arth + PRP; E = Arth; F = Arth + MOR; G = Arth + LA; H = Arth + MA; I = Arth + TRA; J = Arth + SAL; K = Arth + GC; L = Arth + GLU; M = Arth + GLU + LA; N = Arth + NS. In Groups C and D: A = Arth + HA; B = Arth + I-PRF; C = Arth + PDGF; D = Arth + PRP; E = Arth.

## 4. Discussion

In this study, we conducted a comprehensive network meta-analysis comparing the efficacy of various intra-articular drug injection after joint puncture in TMD patients with restricted mouth opening. A total of 38 studies were included, encompassing 13 different drug interventions and 1533 patients diagnosed with TMD. Our findings demonstrated that the I-PRF injection after arthrocentesis significantly improved MMO, as well as lateral and protrusive movement. Additionally, MA injection after arthrocentesis significantly reduced the pain of patients, followed by l-PRF injection. Notably, l-PRF was superior to PRP in functional recovery (MD = 12.85, 95% CI: 6.24–19.46), which may be attributed to PRF’s fibrin matrix that allows for the sustained release of growth factors (*e.g.*, TGF-β1 (Transforming growth factor-β1), PDGF). In contrast, PRP’s therapeutic effects are often short-lived due to its rapid degradation and lower retention at the injection site, as previously demonstrated in biomechanical studies [12]. Similarly, the borderline significance of MA over glucocorticoids (GC) in pain reduction (MD = −2.42, 95% CI: −4.89 to 0.05) aligns with evidence that GCs exert short-term anti-inflammatory effects, whereas MA’s stromal vascular fraction promotes tissue regeneration through paracrine signaling [33]. Contrary to expectations, hyaluronic acid (HA) underperformed against PRF despite its widespread clinical use. This discrepancy may reflect HA’s primary mechanism of action as a visco supplement for joint lubrication rather than a regenerative agent. Previous research has also highlighted the limited long-term benefits of HA in the treatment of TMJ osteoarthritis [43]. Taken together, our findings suggest that I-PRF represents the most promising intervention currently available for improving both functional mobility and symptom relief in patients with TMD undergoing arthrocentesis. The statistical results indicate that intra-articular injection of I-PRF effectively improved both MMO and VAS of TMJ. I-PRF promoted collagen synthesis and released TGF-β1 and platelet-derived growth factors for wound healing, without anticoagulants [44]. In a clinical study by Mohi Eldin *et al*. [45], the use of I-PRF and PRP in sacroiliac joint dysfunction was compared. Patients treated with I-PRF experienced greater pain reduction than those treated with PRP [45]. Several studies to date have evaluated the efficacy of intra-articular I-PRF injection as an adjunct to arthrocentesis in patients with internal TMD. These studies consistently reported positive effects of I-PRF on managing local pain and improving mouth opening limitations [25, 46, 47]. For instance, Albilia *et al*. [48] treated 48 TMJ cases in 37 patients with intra-articular I-PRF injection and reported that 33 TMJs (69%) responded positively to the treatment, showing significant pain relief and dysfunction improvement [42]. Consistent with our findings, I-PRF injection after arthrocentesis in TMD patients is superior to joint puncture alone in reducing pain and improving joint function. Moreover, our findings confirmed that I-PRF injection after joint puncture is superior to PRP injection after arthrocentesis for reducing pain and improving joint function in TMD patients. This suggests that clinicians should consider I-PRF as a preferable option when applying autologous products for intra-articular injections.

In this study, MA injection after arthrocentesis performed well in relieving TMD patients’ pain. The pericellular matrix-rich microenvironment provided by MA tissue possesses substantial regenerative potential. Studies have shown that bone marrow mesenchymal stem cells from adipose tissue secrete many molecules that initiate and maintain angiogenesis, anti-fibrosis, anti-apoptosis, antibacterial and immunomodulatory activities [49]. Malanga *et al*. [50] reported that intra-articular injection of adipose-derived mesenchymal stem cells significantly improved both pain and function in patients with knee osteoarthritis. Sembronio *et al*. [33] proposed that MA injection resulted in superior pain relief and functional recovery compared to standard therapies. Our results found that MA injection after arthrocentesis was significantly superior to joint puncture alone in reducing pain in TMD patients. However, further investigations with larger sample sizes and extended follow-up periods are warranted to evaluate the long-term clinical stability and functional benefits of MA in the treatment of TMJ disorders.

In short, this study underscores the therapeutic potential of autologous biological agents in TMD management, as evidenced by the robust synthesis of 38 randomized trials encompassing 1533 patients. The findings indicate that post-arthrocentesis PRF and MA injections significantly improve jaw mobility and alleviate pain. This systematic mapping of current evidence highlights a paradigm shift toward with PRF emerging as the top-ranked intervention for functional recovery and MA showing exceptional promise in pain modulation.

Looking forward, future research should prioritize multicenter, longitudinal studies to validate the sustained clinical efficacy of these interventions, particularly for MA, where long-term efficacy remains underexplored. Mechanistic investigations into the anti-inflammatory and regenerative properties of autologous products are warranted to optimize their therapeutic use. Furthermore, standardization of injection protocols, dose-response evaluations, and subgroup analyses (*e.g.*, age, disease severity) will be critical in refining personalized treatment strategies and enhancing clinical translation.

## 5. Strengths and limitations

Despite our rigorous adherence to Cochrane systematic review standards and the application of ROB tool in selecting RCTs, this study is not without limitations. First, although stringent inclusion and exclusion criteria improved internal validity, they may have compromised external validity, thereby raising concerns about the generalizability of our findings in broader clinical settings. Second, as with other comparative studies, we encountered unavoidable heterogeneity during the initial inclusion of original studies. Variations such as differences in participant demographics—particularly gender distribution—may have introduced confounding factors that limit the comparability of outcomes across studies. Third, potential biases in the included RCTs may have influenced the overall results. For instance, 18% of studies exhibited a high risk of bias in allocation concealment (*e.g.*, Akhilesh Kumar Singh 2019 lacked description of allocation methods), while 13% had unblinded outcome assessors (*e.g.*, Jose-Maria Oliveras-Moreno 2008), potentially introducing performance and detection biases. Although sensitivity analyses excluding high-risk studies (*e.g.*, Sha-Sha Liu 2023) demonstrated robustness, residual bias from open-label designs or incomplete outcome data (*e.g.*, Songül Cömert Kilic 2015 reported high attrition rates) may still have impacted the reliability of pooled estimates. These methodological discrepancies underscore the need for future trials to adopt stricter blinding and allocation protocols. Finally, with the limited number of studies and the absence of direct comparative data on the interventions, it is imperative to conduct a multicenter, long-term RCT with a larger sample size to further validate the robustness of our findings.

## 6. Conclusions

Based on our findings, we recommend the use of platelet-rich fibrin (PRF) injections following arthrocentesis for patients seeking to improve temporomandibular joint (TMJ) mobility, and micro-fragmented adipose tissue (MA) for those prioritizing pain relief. Overall, intra-articular injection of PRF after joint puncture appears highly effective in enhancing TMJ motor function and alleviating pain in patients with temporomandibular disorders (TMD). However, further high-quality, large-scale studies are warranted to substantiate these conclusions and guide clinical decision-making with greater confidence.

## Supplementary Material

Supplementary material associated with this article can be found, in the online version, at https://files.jofph.com/files/article/1961346825762816000/attachment/Supplementary%20material.zip.




