# Supplementary material for: Effect of intraarticular drug injection in patients with temporomandibular joint disorders with limited mouth opening: a system review and network meta-analysis
Source: J Oral Facial Pain Headache. 2025 Dec 12;39(4):85–99. doi: 10.22514/jofph.2025.041 (PMC12727178; doi:10.22514/jofph.2025.041)
Supplement: Supplementary file 2 [file Supplementary-Tables.docx]

Supplementary material

Supplementary Table 1. Consistency test for MMO.

|  | Coefficient | Standard error | *z* | *p* > \|*z*\| | [95% Confidence interval] |
| --- | --- | --- | --- | --- | --- |
| B *vs.* CON | 15.79224 | 3.545063 | 4.45 | <0.001 | 8.844042–22.74043 |
| C *vs.* CON | 0.8291017 | 3.660211 | 0.23 | 0.821 | −6.34478–8.002984 |
| D *vs.* CON | 2.859096 | 1.999626 | 1.43 | 0.153 | −1.060099–6.77829 |
| E *vs.* CON | 0.0030958 | 1.775824 | 0 | 0.999 | −3.477455–3.483647 |
| F *vs.* CON | 1.803095 | 6.17975 | 0.29 | 0.77 | −10.30899–13.91518 |
| G *vs.* CON | 0.2841723 | 6.44248 | 0.04 | 0.965 | −12.34286–12.9112 |
| H *vs.* CON | 3.700001 | 5.435596 | 0.68 | 0.496 | −6.953572–14.35357 |
| I *vs.* CON | *−*1.196905 | 6.26333 | *−*0.19 | 0.848 | −13.47281–11.079 |
| J *vs.* CON | *−*1.315703 | 3.594924 | *−*0.37 | 0.714 | −8.361625–5.730218 |
| K *vs.* CON | *−*0.5592977 | 2.164853 | *−*0.26 | 0.796 | −4.802332–3.683736 |
| L *vs.* CON | *−*3.530353 | 5.356655 | *−*0.66 | 0.510 | −14.0292–6.968498 |
| M *vs.* CON | *−*0.3558657 | 8.270016 | *−*0.04 | 0.966 | −16.5648–15.85307 |
| N *vs.* CON | 2.463094 | 5.755751 | 0.43 | 0.669 | −8.81797–13.74416 |

Note: CON = Arth + HA; B = Arth + I-PRF; C = Arth + PDGF; D = Arth + PRP; E = Arth; F = Arth + MOR; G = Arth + LA; H = Arth + MA; I = Arth + TRA; J = Arth + SAL; K = Arth + GC; L = Arth + GLU; M = Arth + GLU + LA; N = Arth + NS.

**Supplementary Table 2. League table on MMO.**

| Arth + I-PRF | Arth + PRP | Arth + MA | Arth + NS | Arth + MOR | Arth + PDGF | Arth + LA | Arth + GLU + LA | Arth | Arth + HA | Arth + TRA | Arth + GC | Arth + SAL | Arth + GLU |
| --- | --- | --- | --- | --- | --- | --- | --- | --- | --- | --- | --- | --- | --- |
| Arth + I-PRF | −12.85 (−19.46, −6.24) | −12.26 (−24.92, 0.40) | −13.38 (−25.75, −1.01) | −14.04 (−27.17, −0.91) | −15.12 (−24.98, −5.26) | −15.64 (−29.78, −1.49) | −16.28 (−33.66, 1.11) | −15.84 (−22.08, −9.60) | −15.96 (−22.87, −9.04) | −17.04 (−30.32, −3.76) | −16.44 (−23.74, −9.14) | −17.24 (−26.80, −7.67) | −19.45 (−31.76, −7.14) |
| 12.85 (6.24, 19.46) | Arth + PRP | 0.59 (−10.71, 11.89) | −0.53 (−11.81, 10.75) | −1.19 (−13.30, 10.92) | −2.27 (−10.33, 5.79) | −2.79 (−15.79, 10.21) | −3.43 (−19.90, 13.04) | −2.99 (−6.62, 0.64) | −3.11 (−7.01, 0.79) | −4.19 (−16.47, 8.08) | −3.59 (−8.44, 1.26) | −4.39 (−12.15, 3.38) | −6.60 (−17.57, 4.37) |
| 12.26 (−0.40, 24.92) | −0.59 (−11.89, 10.71) | Arth + MA | −1.12 (−16.56, 14.32) | −1.78 (−17.84, 14.28) | −2.86 (−15.64, 9.92) | −3.38 (−19.82, 13.06) | −4.02 (−23.32, 15.28) | −3.58 (−14.74, 7.57) | −3.70 (−14.30, 6.90) | −4.78 (−20.96, 11.40) | −4.18 (−15.59, 7.23) | −4.98 (−17.69, 7.74) | −7.19 (−22.08, 7.70) |
| 13.38 (1.01, 25.75) | 0.53 (−10.75, 11.81) | 1.12 (−14.32, 16.56) | Arth + NS | −0.66 (−16.39, 15.07) | −1.74 (−14.98, 11.50) | −2.26 (−18.93, 14.42) | −2.90 (−22.40, 16.61) | −2.46 (−13.14, 8.22) | −2.58 (−13.81, 8.65) | −3.66 (−19.52, 12.20) | −3.06 (−14.47, 8.35) | −3.86 (−16.87, 9.16) | −6.07 (−21.22, 9.08) |
| 14.04 (0.91, 27.17) | 1.19 (−10.92, 13.30) | 1.78 (−14.28, 17.84) | 0.66 (−15.07, 16.39) | Arth + MOR | −1.08 (−15.04, 12.88) | −1.60 (−18.85, 15.65) | −2.24 (−22.23, 17.76) | −1.80 (−13.35, 9.75) | −1.92 (−13.98, 10.14) | −3.00 (−14.60, 8.60) | −2.40 (−14.63, 9.84) | −3.20 (−16.94, 10.55) | −5.41 (−21.19, 10.37) |
| 15.12 (5.26, 24.98) | 2.27 (−5.79, 10.33) | 2.86 (−9.92, 15.64) | 1.74 (−11.50, 14.98) | 1.08 (−12.88, 15.04) | Arth + PDGF | −0.52 (−13.96, 12.93) | −1.16 (−17.98, 15.67) | −0.72 (−8.55, 7.11) | −0.84 (−7.98, 6.30) | −1.92 (−16.02, 12.18) | −1.32 (−9.35, 6.71) | −2.12 (−10.61, 6.37) | −4.33 (−15.83, 7.16) |
| 15.64 (1.49, 29.78) | 2.79 (−10.21, 15.79) | 3.38 (−13.06, 19.82) | 2.26 (−14.42, 18.93) | 1.60 (−15.65, 18.85) | 0.52 (−12.93, 13.96) | Arth + LA | −0.64 (−10.75, 9.47) | −0.20 (−13.01, 12.61) | −0.32 (−12.89, 12.24) | −1.40 (−18.77, 15.96) | −0.80 (−13.48, 11.87) | −1.60 (−12.03, 8.83) | −3.81 (−16.81, 9.18) |
| 16.28 (−1.11, 33.66) | 3.43 (−13.04, 19.90) | 4.02 (−15.28, 23.32) | 2.90 (−16.61, 22.40) | 2.24 (−17.76, 22.23) | 1.16 (−15.67, 17.98) | 0.64 (−9.47, 10.75) | Arth + GLU + LA | 0.44 (−15.88, 16.75) | 0.32 (−15.81, 16.45) | −0.76 (−20.86, 19.33) | −0.16 (−16.37, 16.05) | −0.96 (−15.48, 13.56) | −3.17 (−19.64, 13.29) |
| 15.84 (9.60, 22.08) | 2.99 (−0.64, 6.62) | 3.58 (−7.57, 14.74) | 2.46 (−8.22, 13.14) | 1.80 (−9.75, 13.35) | 0.72 (−7.11, 8.55) | 0.20 (−12.61, 13.01) | −0.44 (−16.75, 15.88) | Arth | −0.12 (−3.58, 3.35) | −1.20 (−12.93, 10.53) | −0.60 (−4.62, 3.42) | −1.40 (−8.84, 6.05) | −3.61 (−14.35, 7.13) |
| 15.96 (9.04, 22.87) | 3.11 (−0.79, 7.01) | 3.70 (−6.90, 14.30) | 2.58 (−8.65, 13.81) | 1.92 (−10.14, 13.98) | 0.84 (−6.30, 7.98) | 0.32 (−12.24, 12.89) | −0.32 (−16.45, 15.81) | 0.12 (−3.35, 3.58) | Arth + HA | −1.08 (−13.31, 11.15) | −0.48 (−4.71, 3.74) | −1.28 (−8.29, 5.74) | −3.49 (−13.94, 6.96) |
| 17.04 (3.76, 30.32) | 4.19 (−8.08, 16.47) | 4.78 (−11.40, 20.96) | 3.66 (−12.20, 19.52) | 3.00 (−8.60, 14.60) | 1.92 (−12.18, 16.02) | 1.40 (−15.96, 18.77) | 0.76 (−19.33, 20.86) | 1.20 (−10.53, 12.93) | 1.08 (−11.15, 13.31) | Arth + TRA | 0.60 (−11.80, 13.00) | −0.20 (−14.08, 13.69) | −2.41 (−18.31, 13.49) |
| 16.44 (9.14, 23.74) | 3.59 (−1.26, 8.44) | 4.18 (−7.23, 15.59) | 3.06 (−8.35, 14.47) | 2.40 (−9.84, 14.63) | 1.32 (−6.71, 9.35) | 0.80 (−11.87, 13.48) | 0.16 (−16.05, 16.37) | 0.60 (−3.42, 4.62) | 0.48 (−3.74, 4.71) | −0.60 (−13.00, 11.80) | Arth + GC | −0.80 (−8.00, 6.41) | −3.01 (−13.59, 7.57) |
| 17.24 (7.67, 26.80) | 4.39 (−3.38, 12.15) | 4.98 (−7.74, 17.69) | 3.86 (−9.16, 16.87) | 3.20 (−10.55, 16.94) | 2.12 (−6.37, 10.61) | 1.60 (−8.83, 12.03) | 0.96 (−13.56, 15.48) | 1.40 (−6.05, 8.84) | 1.28 (−5.74, 8.29) | 0.20 (−13.69, 14.08) | 0.80 (−6.41, 8.00) | Arth + SAL | −2.21 (−9.96, 5.53) |
| 19.45 (7.14, 31.76) | 6.60 (−4.37, 17.57) | 7.19 (−7.70, 22.08) | 6.07 (−9.08, 21.22) | 5.41 (−10.37, 21.19) | 4.33 (−7.16, 15.83) | 3.81 (−9.18, 16.81) | 3.17 (−13.29, 19.64) | 3.61 (−7.13, 14.35) | 3.49 (−6.96, 13.94) | 2.41 (−13.49, 18.31) | 3.01 (−7.57, 13.59) | 2.21 (−5.53, 9.96) | Arth + GLU |

League table for outcomes: League table on MMO. Arth: arthrocentesis; HA: hyaluronic acid; I-PRF: liquid platelet rich fibrin; PDGF: platelet-derived growth factor; PRP: platelet rich plasma; MOR: morphine; LA: local anesthetics; MA: Microfragmented Adipose; TRA: tramadol; SAL: saline; GC: glucocorticoid; GLU: glucose; NS: non-steroidal.

Supplementary Table 3. Consistency test for VAS.

|  | Coefficient | Standard error | *z* | *p* > \|*z*\| | [95% Confidence interval] |
| --- | --- | --- | --- | --- | --- |
| B *vs.* CON | −0.9848647 | 0.9974861 | −0.99 | 0.323 | −2.939902–0.9701722 |
| C *vs.* CON | 0.7086522 | 0.866171 | 0.82 | 0.413 | −0.9890117–2.406316 |
| D *vs.* CON | −0.2367432 | 0.4661993 | −0.51 | 0.612 | −1.150477–0.6769906 |
| E *vs.* CON | 0.8533923 | 0.4203717 | 2.03 | 0.042 | 0.0294789–1.677306 |
| F *vs.* CON | −0.5466133 | 1.483279 | −0.37 | 0.712 | −3.453787–2.36056 |
| G *vs.* CON | −6.87305 | 13.27125 | −0.52 | 0.605 | −32.88423–19.13813 |
| H *vs.* CON | −1.8 | 1.39902 | −1.29 | 0.198 | −4.542028–0.9420282 |
| I *vs.* CON | −0.2466133 | 1.568476 | −0.16 | 0.875 | −3.32077–2.827543 |
| J *vs.* CON | 2.514895 | 0.6396578 | 3.93 | <0.001 | 1.261189–3.768602 |
| K *vs.* CON | 0.6204409 | 0.5023085 | 1.24 | 0.217 | −0.3640658–1.604947 |
| L *vs.* CON | 1.654692 | 1.118293 | 1.48 | 0.139 | −0.5371228–3.846507 |
| M *vs.* CON | 1.354681 | 1.792104 | 0.76 | 0.450 | −2.157779–4.86714 |
| N *vs.* CON | 1.209839 | 0.7411151 | 1.63 | 0.103 | −0.2427201–2.662398 |

Note: CON = Arth + HA; B = Arth + I-PRF; C = Arth + PDGF; D = Arth + PRP; E = Arth; F = Arth + MOR; G = Arth + LA; H = Arth + MA; I = Arth + TRA; J = Arth + SAL; K = Arth + GC; L = Arth + GLU; M = Arth + GLU + LA; N = Arth + NS.

**Supplementary Table 4. League table on VAS.**

| Arth + MA | Arth + I-PRF | Arth + LA | Arth + PRP | Arth + MOR | Arth + HA | Arth + TRA | Arth + GC | Arth + PDGF | Arth | Arth + GLU + LA | Arth + NS | Arth + GLU | Arth + SAL |
| --- | --- | --- | --- | --- | --- | --- | --- | --- | --- | --- | --- | --- | --- |
| Arth + MA | 0.82 (−2.55, 4.18) | −5.07 (−31.23, 21.08) | 1.56 (−1.33, 4.45) | 1.25 (−2.74, 5.25) | 1.80 (−0.94, 4.54) | 1.55 (−2.57, 5.67) | 2.42 (−0.49, 5.33) | 2.51 (−0.72, 5.73) | 2.65 (−0.21, 5.52) | 3.15 (−1.30, 7.61) | 3.01 (−0.09, 6.11) | 3.45 (−0.06, 6.97) | 4.31 (1.30, 7.33) |
| −0.82 (−4.18, 2.55) | Arth + PRF | −5.89 (−31.97, 20.19) | 0.75 (−1.11, 2.61) | 0.44 (−2.91, 3.78) | 0.98 (−0.97, 2.94) | 0.74 (−2.75, 4.23) | 1.61 (−0.44, 3.65) | 1.69 (−0.88, 4.27) | 1.84 (−0.01, 3.68) | 2.34 (−1.65, 6.33) | 2.19 (−0.15, 4.54) | 2.64 (−0.26, 5.53) | 3.50 (1.23, 5.77) |
| 5.07 (−21.08, 31.23) | 5.89 (−20.19, 31.97) | Arth + LA | 6.64 (−19.39, 32.66) | 6.33 (−19.84, 32.49) | 6.87 (−19.14, 32.88) | 6.63 (−19.56, 32.81) | 7.49 (−18.52, 33.51) | 7.58 (−18.47, 33.63) | 7.73 (−18.29, 33.75) | 8.23 (−17.96, 34.42) | 8.08 (−17.95, 34.12) | 8.53 (−17.52, 34.57) | 9.39 (−16.59, 35.37) |
| −1.56 (−4.45, 1.33) | −0.75 (−2.61, 1.11) | −6.64 (−32.66, 19.39) | Arth + PRP | −0.31 (−3.22, 2.60) | 0.24 (−0.68, 1.15) | −0.01 (−3.09, 3.07) | 0.86 (−0.29, 2.00) | 0.95 (−0.97, 2.86) | 1.09 (0.24, 1.94) | 1.59 (−2.01, 5.19) | 1.45 (−0.17, 3.06) | 1.89 (−0.44, 4.22) | 2.75 (1.27, 4.24) |
| −1.25 (−5.25, 2.74) | −0.44 (−3.78, 2.91) | −6.33 (−32.49, 19.84) | 0.31 (−2.60, 3.22) | Arth + MOR | 0.55 (−2.36, 3.45) | 0.30 (−2.27, 2.87) | 1.17 (−1.79, 4.12) | 1.26 (−2.10, 4.61) | 1.40 (−1.39, 4.19) | 1.90 (−2.63, 6.43) | 1.76 (−1.41, 4.92) | 2.20 (−1.40, 5.80) | 3.06 (−0.06, 6.18) |
| −1.80 (−4.54, 0.94) | −0.98 (−2.94, 0.97) | −6.87 (−32.88, 19.14) | −0.24 (−1.15, 0.68) | −0.55 (−3.45, 2.36) | Arth + HA | −0.25 (−3.32, 2.83) | 0.62 (−0.36, 1.60) | 0.71 (−0.99, 2.41) | 0.85 (0.03, 1.68) | 1.35 (−2.16, 4.87) | 1.21 (−0.24, 2.66) | 1.65 (−0.54, 3.85) | 2.51 (1.26, 3.77) |
| −1.55 (−5.67, 2.57) | −0.74 (−4.23, 2.75) | −6.63 (−32.81, 19.56) | 0.01 (−3.07, 3.09) | −0.30 (−2.87, 2.27) | 0.25 (−2.83, 3.32) | Arth + TRA | 0.87 (−2.25, 3.99) | 0.96 (−2.54, 4.46) | 1.10 (−1.86, 4.06) | 1.60 (−3.04, 6.24) | 1.46 (−1.86, 4.78) | 1.90 (−1.84, 5.64) | 2.76 (−0.51, 6.04) |
| −2.42 (−5.33, 0.49) | −1.61 (−3.65, 0.44) | −7.49 (−33.51, 18.52) | −0.86 (−2.00, 0.29) | −1.17 (−4.12, 1.79) | −0.62 (−1.60, 0.36) | −0.87 (−3.99, 2.25) | Arth + GC | 0.09 (−1.82, 2.00) | 0.23 (−0.75, 1.22) | 0.73 (−2.81, 4.28) | 0.59 (−0.97, 2.15) | 1.03 (−1.21, 3.28) | 1.89 (0.55, 3.24) |
| −2.51 (−5.73, 0.72) | −1.69 (−4.27, 0.88) | −7.58 (−33.63, 18.47) | −0.95 (−2.86, 0.97) | −1.26 (−4.61, 2.10) | −0.71 (−2.41, 0.99) | −0.96 (−4.46, 2.54) | −0.09 (−2.00, 1.82) | Arth + PDGF | 0.14 (−1.72, 2.01) | 0.65 (−3.15, 4.45) | 0.50 (−1.69, 2.69) | 0.95 (−1.68, 3.57) | 1.81 (−0.11, 3.72) |
| −2.65 (−5.52, 0.21) | −1.84 (−3.68, 0.01) | −7.73 (−33.75, 18.29) | −1.09 (−1.94, −0.24) | −1.40 (−4.19, 1.39) | −0.85 (−1.68, −0.03) | −1.10 (−4.06, 1.86) | −0.23 (−1.22, 0.75) | −0.14 (−2.01, 1.72) | Arth | 0.50 (−3.07, 4.07) | 0.36 (−1.15, 1.86) | 0.80 (−1.48, 3.08) | 1.66 (0.26, 3.06) |
| −3.15 (−7.61, 1.30) | −2.34 (−6.33, 1.65) | −8.23 (−34.42, 17.96) | −1.59 (−5.19, 2.01) | −1.90 (−6.43, 2.63) | −1.35 (−4.87, 2.16) | −1.60 (−6.24, 3.04) | −0.73 (−4.28, 2.81) | −0.65 (−4.45, 3.15) | −0.50 (−4.07, 3.07) | Arth + GLU + LA | −0.14 (−3.85, 3.56) | 0.30 (−2.44, 3.04) | 1.16 (−2.12, 4.44) |
| −3.01 (−6.11, 0.09) | −2.19 (−4.54, 0.15) | −8.08 (−34.12, 17.95) | −1.45 (−3.06, 0.17) | −1.76 (−4.92, 1.41) | −1.21 (−2.66, 0.24) | −1.46 (−4.78, 1.86) | −0.59 (−2.15, 0.97) | −0.50 (−2.69, 1.69) | −0.36 (−1.86, 1.15) | 0.14 (−3.56, 3.85) | Arth + NS | 0.44 (−2.04, 2.93) | 1.31 (−0.41, 3.02) |
| −3.45 (−6.97, 0.06) | −2.64 (−5.53, 0.26) | −8.53 (−34.57, 17.52) | −1.89 (−4.22, 0.44) | −2.20 (−5.80, 1.40) | −1.65 (−3.85, 0.54) | −1.90 (−5.64, 1.84) | −1.03 (−3.28, 1.21) | −0.95 (−3.57, 1.68) | −0.80 (−3.08, 1.48) | −0.30 (−3.04, 2.44) | −0.44 (−2.93, 2.04) | Arth + GLU | 0.86 (−0.94, 2.66) |
| −4.31 (−7.33, −1.30) | −3.50 (−5.77, −1.23) | −9.39 (−35.37, 16.59) | −2.75 (−4.24, −1.27) | −3.06 (−6.18, 0.06) | −2.51 (−3.77, −1.26) | −2.76 (−6.04, 0.51) | −1.89 (−3.24, −0.55) | −1.81 (−3.72, 0.11) | −1.66 (−3.06, −0.26) | −1.16 (−4.44, 2.12) | −1.31 (−3.02, 0.41) | −0.86 (−2.66, 0.94) | Arth + SAL |

League table for outcomes: League table on VAS. Arth: arthrocentesis; HA: hyaluronic acid; I-PRF: liquid platelet rich fibrin; PDGF: platelet-derived growth factor; PRP: platelet rich plasma; MOR: morphine; LA: local anesthetics; MA: Microfragmented Adipose; TRA: tramadol; SAL: saline; GC: glucocorticoid; GLU: glucose; NS: non-steroidal.

Supplementary Table 5. Consistency test for LM.

|  | Coefficient | Standard error | *z* | *p* > \|*z*\| | [95% Confidence interval] |
| --- | --- | --- | --- | --- | --- |
| B *vs.* CON | 0.9065422 | 0.3037414 | 2.98 | 0.003 | 0.3112201–1.501864 |
| C *vs.* CON | 0.325951 | 0.1486193 | 2.19 | 0.028 | 0.0346625–0.6172394 |
| D *vs.* CON | 0.0170027 | 0.2547245 | 0.07 | 0.947 | −0.4822481–0.5162534 |
| E *vs.* CON | 0.0736291 | 0.7418986 | 0.1 | 0.921 | −1.380465–1.527724 |

Note: CON = Arth + HA; B = Arth + I-PRF; C = Arth + PDGF; D = Arth + PRP; E = Arth.

**Supplementary Table 6. League table on LM.**

| Arth + I-PRF | Arth + PDGF | Arth | Arth + PRP | Arth + HA |
| --- | --- | --- | --- | --- |
| Arth + I-PRF | −0.58 (−1.21, 0.05) | −0.83 (−2.34, 0.68) | −0.89 (−1.23, −0.55) | −0.91 (−1.50, −0.31) |
| 0.58 (−0.05, 1.21) | Arth + PDGF | −0.25 (−1.73, 1.22) | −0.31 (−0.85, 0.23) | −0.33 (−0.62, −0.03) |
| 0.83 (−0.68, 2.34) | 0.25 (−1.22, 1.73) | Arth | −0.06 (−1.53, 1.42) | −0.07 (−1.53, 1.38) |
| 0.89 (0.55, 1.23) | 0.31 (−0.23, 0.85) | 0.06 (−1.42, 1.53) | Arth + PRP | −0.02 (−0.52, 0.48) |
| 0.91 (0.31, 1.50) | 0.33 (0.03, 0.62) | 0.07 (−1.38, 1.53) | 0.02 (−0.48, 0.52) | Arth + HA |

League table for outcomes: League table on LM. Arth: arthrocentesis; HA: hyaluronic acid; I-PRF: liquid platelet rich fibrin; PDGF: platelet-derived growth factor; PRP: platelet rich plasma.

Supplementary Table 7. Consistency test for PM.

|  | Coefficient | Standard error | *z* | *p* > \|*z*\| | [95% Confidence interval] |
| --- | --- | --- | --- | --- | --- |
| B *vs.* CON | 1.342588 | 0.3371038 | 3.98 | <0.001 | 0.6818767–2.003299 |
| C *vs.* CON | 0.3623841 | 0.2239075 | 1.62 | 0.106 | −0.0764666–0.8012347 |
| D *vs.* CON | 0.4212298 | 0.2910364 | 1.45 | 0.148 | −0.149191–0.9916506 |
| E *vs.* CON | 0.0658324 | 0.5122975 | 0.13 | 0.898 | −0.9382521–1.069917 |

Note: CON = Arth + HA; B = Arth + I-PRF; C = Arth + PDGF; D = Arth + PRP; E = Arth.

**Supplementary Table 8.** **League table on PM.**

| Arth + I-PRF | Arth + PDGF | Arth + PRP | Arth | Arth + HA |
| --- | --- | --- | --- | --- |
| Arth + I-PRF | −0.29 (−2.47, 1.89) | −0.29 (−2.79, 2.21) | −0.39 (−2.92, 2.14) | −0.65 (−3.20, 1.90) |
| 0.29 (−1.89, 2.47) | Arth + PDGF | −0.00 (−1.09, 1.09) | −0.10 (−1.38, 1.18) | −0.69 (−2.93, 1.55) |
| 0.29 (−2.21, 2.79) | 0.00 (−1.09, 1.09) | Arth + PRP | −0.10 (−1.64, 1.44) | 0.13 (−1.11, 1.37) |
| 0.39 (−2.14, 2.92) | 0.10 (−1.18, 1.38) | 0.10 (−1.44, 1.64) | Arth | −0.40 (−0.90, 0.10) |
| 0.65 (−1.90, 3.20) | 0.69 (−1.55, 2.93) | −0.13 (−1.37, 1.11) | 0.40 (−0.10, 0.90) | Arth + HA |

League table for outcomes: League table on PM. Arth: arthrocentesis; HA: hyaluronic acid; I-PRF: liquid platelet rich fibrin; PDGF: platelet-derived growth factor; PRP: platelet rich plasma.
